# The effects of business practices, licensing, and intellectual property on development and dissemination of the polymerase chain reaction: case study

**DOI:** 10.1186/1747-5333-1-7

**Published:** 2006-07-03

**Authors:** Joe Fore, Ilse R Wiechers, Robert Cook-Deegan

**Affiliations:** 1 Center for Genome Ethics, Law & Policy, Institute for Genome Sciences & Policy, Duke University, Box 90141, Durham, NC 27708, USA; 2 Massachusetts General/McLean Hospital Adult Psychiatry Residency Program, 55 Fruit Street Wang 812, Boston, MA 02114, USA

## Abstract

**Introduction:**

Polymerase chain reaction (PCR) was a seminal genomic technology discovered, developed, and patented in an industry setting. Since the first of its core patents expired in March, 2005, we are in a position to view the entire lifespan of the patent, examining how the intellectual property rights have impacted its use in the biomedical community. Given its essential role in the world of molecular biology and its commercial success, the technology can serve as a case study for evaluating the effects of patenting biological research tools on biomedical research.

**Case description:**

Following its discovery, the technique was subjected to two years of in-house development, during which issues of inventorship and publishing/patenting strategies caused friction between members of the development team. Some have feared that this delay impeded subsequent research and may have been due to trade secrecy or the desire for obtaining lucrative intellectual property rights. However, our analysis of the history indicates that the main reasons for the delay were benign and were primarily due to difficulties in perfecting the PCR technique. Following this initial development period, the technology was made widely available, but was subject to strict licensing terms and patent protection, leading to an extensive litigation history.

**Discussion and evaluation:**

PCR has earned approximately $2 billion in royalties for the various rights-holders while also becoming an essential research tool. However, using citation trend analysis, we are able to see that PCR's patented status did not preclude it from being adopted in a similar manner as other non-patented genomic research tools (specifically, pBR322 cloning vector and Maxam-Gilbert sequencing).

**Conclusion:**

Despite the heavy patent protection and rigid licensing schemes, PCR seems to have disseminated so widely because of the practices of the corporate entities which have controlled these patents, namely through the use of business partnerships and broad corporate licensing, adaptive licensing strategies, and a "rational forbearance" from suing researchers for patent infringement. While far from definitive, our analysis seems to suggest that, at least in the case of PCR, patenting of genomic research tools need not impede their dissemination, if the technology is made available through appropriate business practices.

## Introduction

Over the past two decades, there has been increasing concern that patents on biological tools could impede their dissemination and use in the research community. In an attempt to examine the extent to which these concerns might be valid, we sought to explore a number of case histories of seminal genomic technologies and study how intellectual property concerns, licensing, litigation, and business practices affected their development and dissemination. Our work with the polymerase chain reaction (PCR) seems to present a particularly interesting case study for analyzing these potential impediments to access by researchers. Because PCR, unlike most other early biological research tools, was developed in a corporate setting, its development was subjected to considerations of patenting and licensing from its very inception.

The technology was also protected by robust patent rights and heavily licensed, earning large profits for the entities which have controlled the patents. And yet, PCR has managed to disseminate broadly in the molecular biology world, becoming an indispensable research tool employed in nearly every biological field. Since the core PCR patents began to expire in March, 2005, we are poised to view the entire "lifespan" of the patents and assess how the patenting and business considerations affected the technology throughout its entire 20 year trajectory.

Overall, our study finds that intellectual property rights and licensing have not prevented PCR from being widely adopted and played little role in the initial period of secrecy during its initial in-house development stage. This comes as a result of deliberate business practices on the part of the patent holders, which have given researchers broad access to the technology.

## Case description

### Discovery and development in an industry setting

The history of PCR provides an interesting foil to the traditional development of biological research tools, which, throughout the 1970s and early 80s occurred typically in the academic environment. This case study incorporates many elements not traditionally found in the realm of university research.

The industrial culture at Cetus contributed greatly to the emergence of PCR. The eclectic environment afforded the research team at Cetus the opportunity to take advantage of a wide variety of experts and technical backgrounds, all of which would be crucial to the development of the technology. The commercial nature of the company also granted researchers the flexibility to work on developing PCR, while allowing Cetus to capitalize financially on its lucrative applications.

The norms of proprietary research required a business plan which consisted of perfecting the technological methods, acquiring intellectual property protection, and only then considering other forms of public disclosure, including publication. This stands in stark contrast to the traditional academic model, in which openness and dissemination were foremost, with patents or licensing revenue secondary. As a result of this commercial mindset, it can be argued that PCR did not initially disseminate as quickly through the scientific community as it would have if discovered at an academic institution. Once publicly announced, however, the company had an incentive to find many uses, so long as such uses contributed revenue streams, or were at least revenue neutral. Despite such theoretical concerns over intellectual property rights, most of the reasons for the two-year delay between PCR's discovery and patenting/public disclosure are rather benign and not the result of a deliberate strategy to maintain trade secrecy, except to the extent that the invention had to remain private until a patent application was filed, in order to preserve worldwide patent rights.

However, the rigors of the business environment also threatened the existence of PCR, as management initially viewed its development as diverting resources away from the company's main business objectives. Thus, at several points during its development, those working on the technology were forced to demonstrate not only its technical feasibility, but also its profitability. Unlike researchers working in an academic setting, showing that PCR worked as a basic research tool was not enough; it had to be made applicable to a field relevant to Cetus's business plan, primarily through human diagnostics.

#### Discovery

The initial discovery of the polymerase chain reaction technique is attributed to Kary Mullis, a chemist who won the Nobel Prize in 1993 for his work on PCR. Mullis found himself at Cetus in 1979 after studying chemistry at Georgia Institute of Technology, biochemistry at UC Berkeley, and doing post-doctoral work in pharmaceutical chemistry at UC San Francisco. His work at Cetus focused on synthesizing oligonucleotides, short segments of DNA usually less than 20 nucleotide bases in length, for use by other researchers within the company. He became head of the DNA synthesis lab in 1981, and automated the production of oligonucleotides, making the process of DNA synthesis much less laborious. With productivity on the rise, Mullis remembers, "we nucleotide chemists found ourselves successfully underemployed. Laboratory machines, which we loaded and watched, were making almost more oligonucleotides than we had room for in the freezer...in my laboratory at Cetus, there was a fair amount of time available to think and to putter [1]."

Mullis began experimenting with oligonucleotides and the denaturation/renaturation properties of DNA. Using computer programs, he attempted to quantify the effects of time and temperature on double-stranded DNA, a poorly-understood subject at the time [1]. Mullis made extensive use of iterative loops, a process involving repeated calculation of a formula where the result of each calculation becomes the input for the next calculation. This work showed Mullis that iterative exponential growth could be a very powerful tool for generating large numbers quickly. With the combination of these two ideas: understanding of the complex denaturation/renaturation properties of DNA and the concept of the iterative loop, the stage was set for the breakthrough.

According to Mullis, that breakthrough moment happened one April night in 1983 on the long, winding road from San Francisco to Mendicino [1]. It was on this drive that Mullis brought ideas together for a technique that he envisioned would allow scientists to create billions of copies of a segment of DNA in a matter of hours. After thinking through the relatively simple process, Mullis was convinced that, for some reason, it would not work and it was too simple not to have been attempted. He recalls thinking that "someone, somewhere, must have tried this idea already. Thousands of investigators had, for various reasons, extended single oligonucleotides with polymerases; surely someone would have noticed the possibility of a polymerase chain reaction [1]." Mullis returned to the lab at Cetus and after exhaustive searching, found nothing in the literature to suggest something like a polymerase chain reaction being attempted previously.

However, it would be several months before the PCR concept was tested. Rabinow posits several possible factors that contributed to Mullis' delay: a hectic work schedule at Cetus, a tumultuous love life, and discouraging feedback from colleagues [2]. Mullis asserts that the delay was largely due to two things: the time needed for him to become familiar with the methods and techniques required for PCR and the unwillingness of colleagues to get involved and help with the process [3]. Mullis finally ran his first PCR experiment on September 8, 1983, unsuccessfully attempting to isolate a 400 base pair stretch of the human nerve growth factor gene. His second experiment took place in October, in which he increased the number of cycles of the denaturing/renaturing process, but again to no avail. He decided to change to a simpler target, and switched to using the common cloning vector pBR322. Mullis claims he saw what he believed to be evidence of amplification on December 16, 1983. One night, after several minor modifications to the process, Mullis began to see faint bands on the gel, indicating he had been successful in isolating a 25 base-pair fragments [2].

Upon seeing the first truly successful results, Mullis searched for someone with whom to share the news. He went across the hall to the office of Al Halluin, Cetus's Chief Patent Counsel, who was working late. After viewing the results in the dark room, Halluin congratulated Mullis for his work and began crafting the claims for the patent application. Halluin recalls that he was intrigued by the notion that the PCR process could produce multiple copies of DNA of similar molecular weight, which is analogous to living polymers produced by anionic polymerization [4]. The attorney had filed for patents on such polymers in a previous job and recognized the similar importance. The next day, Halluin continued working on a preliminary draft of the patent application, and presented the idea to Cetus's President, Dr. Robert A. Fildes. As Halluin remembers, Fildes did not share the patent attorney's enthusiasm: "Basically, Bob said, 'You can go ahead and pursue it, but you better not do it at the expense of more important things, like writing patents for therapeutics and cancer and diagnostics [4]." The early interaction between Mullis and Halluin is significant and highlights a key difference between innovation occurring in industry versus academia; not many scientists working in academia have patent attorneys across the hall from their labs. The industry environment clearly places emphasis on commercialization of new technologies from the outset.

Bolstered by what he viewed as promising results, Mullis decided to switch to a larger, more complicated system, working first with the 50,000 base-pair lambda phage and then moving back to human DNA, using a 58 base-pair section of the beta-globin gene. The beta-globin gene was important because it is the site of the mutation that causes sickle-cell anemia, and a source of great interest for Cetus scientists because of its potential for diagnostic tests. In June of 1984, Mullis presented a poster showing PCR amplification of the beta-globin gene at the Cetus annual scientific meeting in Monterey, but it was largely ignored [2].

The summer of 1984 was a time of uncertainty regarding the future of PCR and Mullis at Cetus Corporation. As a start-up company in the fledgling biotechnology industry, Cetus was forced to invest its resources in those projects which seemed most likely to realize commercial success and profitability in the short-term. The company was focusing its efforts on diagnostics, cancer therapeutics, and agriculture [2]. Thus, promoting the development of PCR as a basic research tool was "looked on by top management as requiring resources that might detract from the main goal [5]." There were also several senior researchers who still questioned whether PCR worked as claimed [4]. In addition, Mullis' erratic behavior had been causing problems at work, and separate from its decisions to pursue PCR research, management was trying to decide whether or not to fire Mullis. Despite these obstacles, Mullis was relieved of his duties as head of the DNA synthesis lab and given a one year trial period in which to focus his work on PCR [2].

#### Development and dissemination of PCR

Further development of PCR focused on improving the technique as it applied to diagnostics, which could have immediate commercial value. Until this point, all of the work on PCR was done by Mullis and his lab technician, Fred Faloona. During the summer of 1984, the "PCR group" was formed by adding Henry Erlich, Norman Arnheim, Randy Saiki, Glenn Horn, and Steven Scharf for the purposes of perfecting protocols and developing applications for the technique [2]. For PCR to reach its full potential as a diagnostic and research tool, it would require the effort of a dedicated team of scientists and skilled technicians, along with occasional, reluctant support from the Cetus management. John Sninsky, a senior scientist at Cetus at the time, claims that the development of PCR represents:

"The quintessential use of biotechnology in an industrial setting, because it took advantage of larger, rather than smaller teams of scientists to explore to its fruition an idea. But each member of that team had brought to that team a very different experience, really a multidisciplinary approach, so you had the creative chemistry background that Kary brought to the area...the enthusiasm and intelligence, really, of Fred Faloona...the human genetics background and the careful experimentation in molecular biology that Henry Erlich and Norm Arnheim and their colleagues brought to that [6]."

The group worked throughout the summer and fall to obtain reliable results. An experiment run by Scharf and Faloona on November 14, 1984 provided them with those results [6]. With solid experimental data in hand, the group worked towards perfecting the technique. Arnheim and Erlich designated Saiki as the full-time technician charged with that task. However, with success came questions about how to proceed.

Development of a new technology in the corporate environment required developing not only publication strategies, but also patenting and business strategies.

In the early months of 1985, members of the PCR group along with Tom White, then Cetus's Vice President of Research, visited several large corporations trying to drum up support and investment for the diagnostics program, including potential applications of PCR. During one of their presentations, an audience member asked questions that indicated he understood the idea behind PCR [2]. Furthermore, White heard through mutual friends that Mullis was talking about PCR to people outside of Cetus. It became clear that disclosure of the technology was on the verge of happening, and Cetus needed to ensure that it capitalized on the invention. Given the highly competitive nature of biotechnology, it was decided that the strategy would be to file patents first, and publish second. Halluin states this strategy also took into account the general concern for international patent filings, as they did not want publications to preclude patenting in foreign countries where any previous publications are viewed as prior art [4].

The filing of the first PCR patent application (which would later be split into two U.S. patents numbered 4,683,202 and 4,683,195) occurred on March 28, 1985. According to Halluin, part of the reason for the delay in filing is attributable to the fact that Janet Hasak, the person whom he wanted to complete the drafting of the patent applications, was on maternity leave during most of the summer of 1984 [4]. When Hasak returned to work in September, she and Mullis began drafting the final versions of the patent application. Mullis states he wanted the application to have "broad claims that would not appear shortsighted when derivative technologies started to appear. But I did not want them to wander...I wanted them to say what they had to say in the least number of words [7]." However, in formulating more general claims, Mullis and Hasak failed to include several key concepts, most notably a claim of the situation where the target DNA sequence was imbedded in a larger sequence [7]. Mullis's desire for brevity would come back to haunt Cetus later, as the company would become embroiled in costly legal challenges, which will be discussed in further detail below.

Having taken steps to protect the intellectual property rights for PCR, Cetus now shifted focus to dissemination of the new technology to the research community. While support emerged for publicly disclosing the PCR concept, there was some contention over how best to proceed. At one point, Mullis suggested keeping PCR as an in-house trade secret. Tom White recalls that "Mullis proposed selling tubes containing reagents – the mixture of which would remain a secret – into which one had put DNA." He remembers Mullis insisting that "people would be amazed...at the end when they saw the huge amount of DNA produced [2]." This plan had two major flaws: first, researchers would be able to reverse-engineer the constitutive elements, rendering it unnecessary for them to purchase the materials from Cetus; and, second, some of the patent applications which had been filed in March (those filed abroad, where patent applications are published after 18 months) would become public information, unless the patent applications were abandoned. However, Mullis insists that his comments on trade secrecy were made casually: "it's not like I was going around Cetus proselytizing that idea [3]." Additionally, some members of Cetus management thought that disclosure of PCR in publication would make the technology less attractive to Kodak, who had shown interest in pursuing a diagnostics partnership with Cetus based on PCR [2].

Ultimately, plans were made for Saiki to present PCR diagnostic results at the October 1985 annual meeting of the American Society for Human Genetics. The group also decided to publish two papers: a "theory" paper, written by Mullis, covering the basic concept of PCR as an amplification technique, followed by an "applications" paper demonstrating the technology's use in the human beta-globin system, on which the group had been working [2]. Despite this plan, the team working on the applications paper soon took the lead, confirming their results with radioactive Southern blotting. Saiki recalls Mullis and Faloona "wanted to do more elegant experiments. They didn't want to use radioactivity...So they went off and wanted to do it in a nonradioactive manner, which at the time was very difficult [6]." Mullis states that the final experiment he wanted to run, showing that PCR could be done in a single copy gene in human DNA, turned out to be much harder than he anticipated [3]. Whatever the exact cause of the delay, the fact remained that the applications paper was ready for publication, and the Cetus management pushed for it to be submitted to *Science *in September 1985. The applications paper appeared in the December 20, 1985 issue of *Science*, and acknowledges the use of "the polymerase chain reaction (PCR) procedure of Mullis and Faloona [8]." The paper reflects the continued emphasis of those within Cetus to focus on the commercial applications of PCR (in this case, diagnostic testing) while largely ignoring its potential as a basic research tool.

Publishing the theory paper now proved to be difficult. Mullis submitted the theory paper to *Nature *in December 1985, and it was rejected as merely technical and unoriginal. Hurriedly, Mullis submitted the manuscript to *Science*, this time with an explanatory cover letter stating precisely how the article differed from the previous PCR paper. It was also rejected. The rejections angered Mullis, who felt as though the credit for his invention was stolen from him through the publication of the original *Science *paper [3]. He explained that "the [applications] paper, as it was first sort of drafted, still avoided talking directly to how you did PCR...but by the time it got back and forth between *Science *editors and Norman [Arnheim], it was almost two-thirds of it was PCR. It told you everything you needed to know [6]." The theory paper was finally accepted for publication in *Methods of Enzymology *in May 1985, but through a series of delays, was not seen in print until 1987 [9].

The strategy of patent first, then publish, differs significantly from cases of technologies discovered in an academic setting. For example, Professor Sir Edwin Southern created the technique which bears his name, the Southern blot, while working at the MRC Mammalian Genome Unit in 1973 [10]. During the two years between his discovery and the publication of his work, Southern engaged in a very liberal pre-publication sharing strategy. He literally drew schematics on scraps of paper and gave it to other scientists. As word about this new blotting method began to spread through the research community, Southern allowed colleagues to further disseminate the information, requesting only that he receive acknowledgement for the origination of the process [11], [12]. Cetus' course of action also stands in stark contrast even to patented technologies which were developed in academic settings. One good example is the case of Stanford's Cohen-Boyer patent on recombinant DNA technology, which was patented only after publication and a front-page *New York Times *story heralding the technique [13].

### Commercialization and intellectual property challenges

With the patent applications filed and the scientific papers accepted for publication, the focus of the PCR project shifted to commercialization, making PCR available to researchers and profitable for Cetus. To accomplish these goals, Cetus formed strategic partnerships with other corporations, ensuring that outside entities would be able to contribute their expertise to the further development of the technology.

After spending the first three years in relative obscurity as an in-house project, PCR was ready for its presentation to a worldwide audience. In response to increased attention from outside researchers and companies, Cetus realized that it had to develop adaptable licensing schemes that were receptive to the demands of the scientific community. This new commitment to accessibility coincided with PCR's explosion in popularity, as researchers around the world continued to find still newer applications for the technology.

Eventually, PCR would outgrow Cetus, and the rights were acquired by Swiss pharmaceutical giant Hoffmann-La Roche, a company with sufficient resources to both develop the technology further and increase access for researchers. These efforts helped lead PCR to its current status as an indispensable, nearly ubiquitous biological research tool.

#### Final improvements and PCR's public debut

Before PCR could be fully automated and made truly marketable, the team needed to find a better polymerase enzyme. The PCR process involved heating the DNA to 95 degrees Celsius, which had the effect of denaturing the *E. coli *polymerase enzyme they were using, rendering it inactive for the next round of amplification. Hence, fresh polymerase had to be added to each cycle, causing the process to be slow and laborious. The PCR group tested several different thermophillic bacterial species, and eventually settled on *Thermus aquaticus*, a strain of bacteria which had been discovered living in Yellowstone hot springs. Because the bacteria lived in water that often approached boiling conditions, its polymerase enzyme had no problem remaining functional at the PCR temperatures [6].

Work purifying the polymerase enzyme was led by David Gelfand and Susanna Stoffel, and with three weeks of overtime work, the new *Taq *polymerase was ready to be used in PCR experiments [2]. The thermostable properties of the enzyme also allowed the annealing reactions (when the polymerase enzyme reassembles the separated DNA strands to double the desired sequence) to be carried out at a higher temperature. The work was so significant, that the team would eventually be granted a patent on the discovery (US patent 4,889,818). The switch to *Taq *polymerase had increased the efficacy of PCR and paved the way for full automation via thermal cyclers (instruments designed to heat and cool the reaction mixtures for PCR). In short, all the elements were in place for the technique to become a staple in nearly every molecular biology lab in the world.

Excitement within the research community took off after Mullis had the opportunity to present PCR at the May 1986 symposium at Cold Spring Harbor entitled "The Molecular Biology of Homo Sapiens." Mullis recalls the reception "was definitive...I had invented something they could use. Everyone told me that it was new and that, furthermore, it was a splendid contribution [7]." The reaction from the scientific community was quick and impressive. Halluin remembers that after Mullis' presentation, Cetus began receiving many inquiries about the "PCR thing", causing management to look more closely at the possibilities for further developing the technology [4].

Additionally, since his presentation at Cold Spring Harbor, Mullis was becoming the public face of PCR. This ignited contention over who should receive credit for the invention and development of the technology. Cetus enhanced some team members' fears about loss of recognition in the spring of 1986, when Mullis was given an unprecedented $10,000 bonus as a reward for his work; everyone else in the PCR group was given the traditional sum of one dollar [2].

Inventorship issues and what Mullis perceived as poor treatment from others in the PCR group caused the rift between him and the rest of the Cetus team to grow. In September 1986, the situation reached a boiling point, and Mullis sat down with White to discuss their options. After a brief exchange, they agreed that Mullis would leave the company, receiving five months salary as severance pay [2].

#### Business strategies for the emerging PCR market

As demand for PCR grew, Cetus adopted three distinct business strategies for the three major fields in which PCR could be marketed: human diagnostics, research applications, and forensics [5]. The diagnostics program would follow a "hands-off" approach. The decision was made to license rights to perform diagnostics using PCR technology to other companies. Cetus finalized a partnership with Kodak to create *in vitro *diagnostics in February of 1986. Under the terms of the agreement, Kodak and Cetus would provide 65 percent and 35 percent of the funding, respectively, and would share profits proportionally, as well [14].

Cetus would capitalize on any potential research applications by manufacturing the instruments and reagents through joint ventures. For example, Cetus entered into a joint-venture with Perkins-Elmer to develop diagnostic instruments and reagents for use in biomedical research in December 1985. Perkins-Elmer Cetus Instruments (PECI) was responsible for manufacturing the first DNA thermal cyclers, which automated the PCR process, as well as the necessary buffers, polymerases, and nucleotides required to perform the technique. Under the terms of the arrangement, Cetus held a 49 percent stake in the joint-venture, while Perkins-Elmer controlled 51 percent [15]. The first of these reagents, the "GeneAmp PCR reagent kit" and thermal cyclers were commercially available within two years, hitting the market in November 1987 [5].

This desire for cooperation sprang mostly from necessity. During the mid-1980s, Cetus executives still aspired to become a pharmaceutical company. Since the management wanted to focus resources on the production of drugs and therapeutics, joint enterprises were seen as the only way to fully fund the projects not dedicated to these goals. Sninsky summarized the business plan: "it was felt that rather than being an instrument company, or trying to be a therapeutics company and an instrument company, that we would put in place joint ventures or relationships such that other major players could do the marketing and manufacturing, and we could do the earlier feasibility and research aspects of the studies [6]." Only the forensics applications were viewed as a business that could be operated entirely in-house [5].

By 1986, PCR had caught on with a handful of scientists, particularly in the area of human diagnostics. Half of the papers published in that year using PCR used the technique specifically for diagnostic applications. Soon, the prospects for the PCR diagnostics market were booming; more than 50 different organizations had approached Cetus about licensing PCR for diagnostic purposes [5]. Cetus's senior director of corporate ventures, William Gerber, claimed that after a decade the total PCR diagnostics market would be worth $1.5 billion [16].

In November 1988, with these lofty goals in mind, Cetus announced the creation of an entire division of the company dedicated solely to the development of PCR. This division focused primarily on creating business partnerships and licensing programs. Forming the division was not only a useful administrative move, but also generated publicity and made PCR more visible in the industry, especially to other businesses that Cetus viewed as potential investors in the technology. With the Cetus-Kodak partnership set to expire in December 1988, Cetus began seeking ways to capitalize on PCR's growing popularity, using the technology as a bargaining chip in order to receive more favorable offers from other large co-sponsors, including DuPont, Abbott, and Hoffmann-La Roche [2].

**Figure 1:**
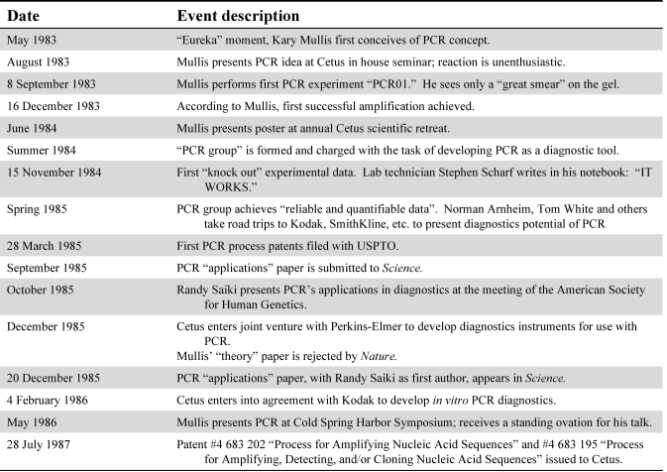
Timeline of key events in the early development of PCR.

Roche represented an interesting partner for Cetus. The Swiss pharmaceutical giant owned the patent rights to recombinant forms of interleukin 2 (IL-2), the production of which was one of Cetus's largest revenue sources [17]. Cetus officials were intrigued at the possibility of swapping rights to PCR technology for rights to manufacture and sell IL-2 without worrying about patent infringement suits. After lengthy negotiations, Cetus announced on January 19, 1989 that the two companies had settled on terms of the agreement: Roche would supply $30 million, paid over five years, to fund diagnostic research. Additionally, Roche would pay a significant royalty on the sale of diagnostic products and services that were developed through the partnership. Roche also agreed to purchase one million shares of Cetus stock. Most importantly, Roche granted Cetus the rights to use the IL-2 patents for commercial purposes without fear of lawsuit [2] (see figure 1).

#### Early challenges to intellectual property rights

In August 1989, the American chemical giant DuPont filed suit against Cetus, alleging the core PCR patents ('202 and '195) were not novel because the processes had been previously described on at least three occasions in the 1970s [18]. DuPont cited work done on the process called "repair replication" by scientists working with Dr. Gobind Khorana at the Massachusetts Institute of Technology [19]. In response to the suit, the U.S. Patent and Trademark Office (USPTO) ordered that both patents' claims be reexamined. Subsequently, the court granted a motion by Cetus to delay the start of the trial until after completion of the reexamination [20]. On August 23, 1990, the USPTO announced its decision to uphold the validity of both patents, rejecting DuPont's claims that any of the papers had fully anticipated PCR. The examiner found the techniques described in the repair replication papers too "indefinite and uncertain" to have made PCR an obvious process. Furthermore, neither of the papers mentioned the possibility of exponential replication, which was a hallmark of the PCR process and was explicitly stated in two claims of the '202 patent [20].

The decision by the USPTO was a major blow to DuPont's case. The court was allowed to consider the reexamination findings along with secondary considerations, including "commercial success of the patent" and "failure of others to perform the invention," as signs that the patent was non-obvious [20]. Given the profitable nature of reagent and thermal cycler sales, and the nearly fifteen year gap between Khorana's original work and the perfection of the PCR technique by the Cetus team, these secondary considerations further tipped the scales in Cetus's favor. Despite strong witnesses on their side, including Dr. Arthur Kornberg from Stanford, who had received the 1959 Nobel Prize for his pioneering work with DNA replication and polymerases, DuPont was not able to prove that PCR had been obvious [7]. On February 28, 1991, after two days of deliberations, the jury issued a verdict in Cetus's favor, echoing the USPTO's previous ruling and unanimously upholding the validity of both the '202 and the '195 patents.

#### PCR outgrows Cetus

**Figure 2:**
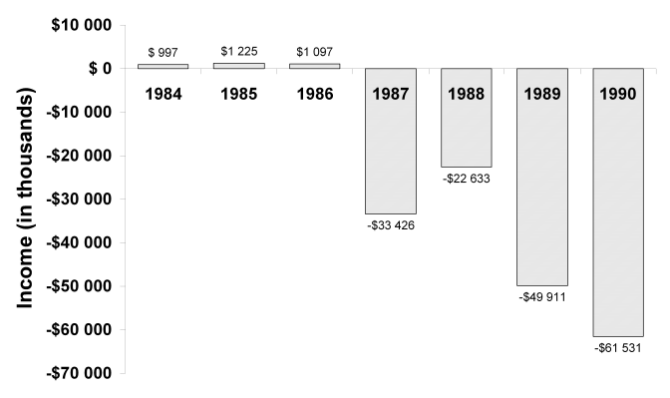
Cetus Corporation income, 1984–1990. All figures in U.S. dollars. Source: Cetus SEC filings 1985–1990.

Meanwhile, outside the courtroom, PCR was becoming an essential research tool in the molecular biology world. In December 1989, *Science *selected PCR as the major scientific development of the year and dubbed the *Taq *enzyme its first annual "Molecule of the Year [21]." The *Science *article highlighted an array of areas where PCR had been introduced, including: diagnostic tests for Lyme disease and AIDS, cancer diagnosis, evolutionary comparison, forensics, paternity determination, matching organ donors and recipients, and uses in DNA sequencing. Along with this explosion in the use of PCR in 1989 came increased profits from PCR reagent kits and thermal cyclers. In that year, net sales for PECI topped $26 million, up from $4 million just two years before. However, even with increased revenue from PCR sales, Cetus was facing financial trouble. The company finished the fiscal year 1990 with a net loss of more than $60 million (See Figure 2) [22]. Cetus was dealt another serious blow when, in July 1990, an FDA review board failed to approve IL-2 for treatment in the United States. The IL-2 program was of crucial significance to the company; according to many at Cetus, President and CEO Bob Fildes had "bet the company" on its success [6]. Following its failure, Fildes left Cetus. With operating losses growing and a key revenue source eliminated, Cetus was in dire straits.

Cetus's management now turned its attention toward Hoffmann-La Roche, hoping to find a purchaser for the bolstered PCR patents. Roche was looking to improve the profitability of its PCR rights. Early in 1991, Roche began a joint-venture with SmithKline Beecham in a deal designed to expand the development of PCR diagnostic testing services [5]. The company then entered into negotiations with Cetus about the possibility of purchasing the PCR franchise outright. The two sides eventually settled on a number: Roche would pay Cetus $300 million in exchange for sole ownership of rights to diagnostic uses of PCR. The deal seemed to be a win-win for both sides. Roche would now control the PCR patents and licensing opportunities for the most lucrative applications, while Cetus would receive much-needed cash to shore up its shaky financial situation. Ron Cape, Cetus's new chairman and CEO commented that, "PCR is worth more to Roche than it is to us...Roche made us an offer we literally could not refuse [23]." Cetus's financial troubles also attracted attention from other biotech firms interested in acquiring the struggling company. On July 23, 1991, Chiron Corporation announced it had agreed to merge with Cetus in a deal estimated at $660 million [23]. The merger was contingent upon the sale of PCR to Roche. The PCR deal was thus a crucial element in Cetus's survival through its merger with Chiron.

However, before the Roche deal or the Chiron merger could take place, Cetus had to deal with another legal challenge. Kodak filed suit against on November 20, 1991, seeking an injunction to stop the transfer of PCR rights to Roche. Kodak claimed that under the terms of their 1986 licensing agreement, neither party could transfer the rights to the PCR-related technologies without the consent of the other [14]. Kodak claimed they would suffer "irreparable injury" by the transfer of the PCR assets, particularly that Roche would receive "trade secrets and know-how that are exclusively licensed to Kodak". The court found Kodak may have suffered some harm from Roche's involvement with Cetus, but the injury had stemmed from the 1989 Cetus-Roche partnership; no further harm was caused by the mere transfer of PCR ownership [14]. Therefore, the motion for injunction was denied, although Kodak was guaranteed a spot at the arbitration hearing regarding new contractual obligations

On December 11, 1991, Hoffmann-La Roche formally acquired the rights to PCR from Cetus for the price of $300 million. Under the terms of the deal, Roche would assume control of all access to PCR technology, although Cetus (now merged with Chiron) would retain the right to use PCR in the development of therapeutics [5]. The PECI joint venture was dissolved and Roche entered into its own alliance with Perkin-Elmer to continue the development of PCR-related instruments [15]. The majority of the Cetus PCR division personnel accepted new positions with Roche, essentially preserving the old research team under new management [6].

#### PCR licensing

The transfer of ownership was integral to the commercial distribution of PCR technology. Cetus focused on applications for diagnostics. Roche planned to expand and commercialize all possible applications of PCR, including its role as a research tool in molecular biology [23]. To help facilitate this, Roche formed a new subsidiary, Roche Molecular Systems, to handle the manufacturing of reagents and control the licensing of PCR rights to other companies [5].

In 1991, over a thousand scientific papers cited one or more of the original PCR publications. Despite the technology's widening use, researchers were increasingly opposed to the old, Cetus licensing scheme. Early in the development of PCR, Cetus had considered the implementation of reach-through licensing agreements (RTLAs), which would have required users to pay Cetus royalties on any invention or marketable product created using PCR technology [24]. This plan was met with harsh criticism from members of the scientific community. An article in *Business Week *compared the arrangement to "a software company demanding royalties from a best-selling author who used its word-processing program [25]." Researchers also decried the high costs of purchasing licensed reagents, like *Taq *polymerase, which cost nearly twice as much as non-licensed products [26].

Roche announced a new, less imposing PCR licensing strategy in January 1992. Roche Molecular stated that the goals of the new policy were to: "1) expand and encourage the use of the technology; 2) derive financial return from the use of the technology by others; and 3) preserve the value of the intellectual property and the patents that were issued on it [24]." Roche created new categories of PCR use, such as research applications and general DNA production, with corresponding royalty and fee schedules. The company also eliminated the $15,000 up-front fee that Cetus had charged to non-profit and academic labs and reduced the royalties on sales of PCR-based products or tests to as low as 9 percent [27].

Roche, furthermore, made it easier to obtain a license to encourage authorized use of the technology. Licenses would now have two parts, an up-front fee component, which could be satisfied simply by purchasing a thermal cycler from an authorized dealer, and a "running royalty" component, which required using licensed reagents to perform PCR [26]. Additionally, Roche expanded the potential uses of the technology by granting licenses for applications in paternity testing and infectious disease diagnostics, two fields for which the company had previously denied granting PCR rights [28]. PCR was now open to more people, for use in more applications, than ever before. Kathy Ordoñez, President of Roche Molecular Systems, proclaimed that the new arrangements would "insure that PCR technology reaches its full potential as rapidly as possible [28]." This move would hasten PCR's diffusion into previously untapped markets, setting the stage for PCR to become *the *standard technology for genetic testing.

### Enforcing the patents

As the PCR market exploded in the early 1990s, so did the temptation to find ways around obtaining proper licenses for running reactions. Despite reducing PCR fees and reducing use restrictions, widespread infringement of the PCR process and product patents forced Roche to aggressively assert its intellectual property rights. The company attempted to monitor researchers who were running reactions with unlicensed reagents or thermal cyclers. However, Roche stopped short of seeking damages from scientists. Instead, the company chose to focus litigation efforts on the companies who were making the infringement possible by selling unauthorized reagents and instruments. The first of these suits was filed just months after Roche gained control of the PCR patents.

#### Roche v. Promega

On October 27, 1992, Roche filed suit in Northern California District Court against Promega Corporation for infringement of patent number 4,889,818 (the '818 patent), which covered the purified n*Taq *polymerase used in the PCR reaction. Two years earlier, Promega and Cetus had entered into a licensing agreement, whereby Promega was granted the right to manufacture, use, and sell *Taq *polymerase only for non-PCR uses. The company was offered the chance to purchase a license which included selling *Taq *for use with PCR. Promega declined to pay five times more for that right, asserting that there was a sufficient market for non-PCR uses of *Taq *polymerase [29]. Under the licensing agreement, Promega paid $30,000 up-front costs and 10 percent royalties on all sales of Taq, and was forbidden from all attempts to "advertise or otherwise promote or directly or indirectly facilitate the use of Licensed Products in PCR Applications [30]."

Roche claimed Promega had violated this portion of the agreement by manufacturing products that were aimed at encouraging customers to use them to perform PCR. Promega had sold more than fifty million units of *Taq *between July 1990 and June 1993. Roche was seeking damages greater than $30 million to recoup the profits that were lost by customers purchasing *Taq *from Promega for use with PCR, instead of from another, fully-licensed company [30]. The court wanted to know who was infringing the PCR patents because of Promega's actions. On May 16, 1995, Roche responded by providing the court with a list of more than two hundred individuals, including researchers working at such elite institutions as the National Cancer Institute, the Howard Hughes Medical Institute, Stanford Medical School, M.I.T., and Harvard University [31]. Roche publicly insisted that it did not intend to sue any of the individuals or institutions on the list, asserting that the fault was Promega's for encouraging the infringement. Dennis Tramaloni, lead counsel for Hoffmann La-Roche summarized the company's position: "Promega has induced researchers engaged in highly practical research to infringe our patents...We're suing the inducer, not suing the many parties who have been induced [32]." While Roche's actions troubled many university researchers, its stance was in line with the traditional corporate practice of "rational forbearance," the overlooking of infringing activity because of the negative stigma of suing non-profit organizations and the high costs of litigation and low damage rewards [33]. Despite the company's assurances, the announcement sent shockwaves through the U.S. research community, as people feared that Roche and other corporations might crack down on "unauthorized" use of basic research tools [34]. Ultimately, however, the company maintained their pledge and declined to bring charges against any individuals or research centers.

Promega argued they could not be guilty of infringement because the '818 patent should never have been granted by the USPTO [35]. Promega formed two arguments to support this assertion: the "invalidity" and the "inequitable conduct" argument. [34] The "invalidity" argument held that Cetus's patent claims were not sufficiently novel to warrant the issuance of a patent. Promega argued that *Taq *enzyme had been previously isolated by Chien and colleagues at the University of Cincinnati [36] and Kaledin and colleagues working in Moscow [37]. In fact, Cetus's initial *Taq *patent application had been denied by the patent examiner, who had concluded the Cetus claims of the purified polymerase were not sufficiently novel from the prior art to warrant a patent. Cetus re-petitioned the examiner, this time successfully obtaining the patent based on further evidence collected through their research, which demonstrated the polymerase isolated by the Chien and Kaledin groups were merely fragments of the entire enzyme [35]. However, Promega presented evidence at trial suggesting Cetus did not perform side-by-side control experiments between their "purified" *Taq *enzyme and the polymerase isolated by the Chien and Kaledin groups, experiments that would have rendered the claims obvious and unpatentable. The court found that Cetus acted with "gross negligence" for failing to perform these crucial experiments [29].

The "inequitable conduct" argument was that Cetus had obtained the patent by intentionally deceiving the USPTO. The court agreed with Promega's claims, finding that Cetus had committed "inequitable conduct" on no fewer than eight separate occasions. The court found that, among other things, Cetus had withheld key experimental data, falsely suggested that they had performed comparative experiments, and overrepresented the purity of the isolated enzyme. On December 7, 1999, after more than seven years of legal battles, Judge Vaughn Walker ruled that the '818 patent covering *nTaq *polymerase was unenforceable [35]. Despite the verdict, Roche maintained that the ruling would have little effect on profits, because 90 percent of the *Taq *polymerase sold was derived from the recombinant form (*rTaq*), which is covered by separate patents that were not affected by the ruling [38].

Roche appealed the ruling, and in March 2003 a U.S. Federal Court of Appeals ruled 2–1 to overturn two of the "acts of misconduct" that Walker had cited in his opinion; the court upheld the other six [39]. The court also ruled that Judge Walker should reevaluate his ruling on whether Cetus's actions were serious enough to warrant revocation of their patents. In May 2004, Judge Walker upheld his original ruling, finding that Cetus's actions were sufficiently egregious to cause invalidation of the '818 patent [40]. Since the original licensing agreement between Roche and Promega was based on the validity of the '818 patent, Roche could not recoup damages for breech of contract [41].

However, the news was not all bad for Roche. Judge Walker did leave the door open for the company to pursue damages from Promega for infringement of the '202 and '195 patents. Promega argued that these claims should be thrown out under the "unclean hands" doctrine, maintaining that Cetus's "unconscionable acts" during the obtaining of the '818 patent tainted their rights to the related PCR patents [41]. The court found these arguments unconvincing, and upheld the validity of the '195 and '202 patents. Litigation in this matter is ongoing.

#### Applera/Roche v. MJ research

In June 1998, when Roche brought suit against instrument manufacturer MJ Research, the company was joined by Applera at the plaintiff's table. MJ had produced thermal cyclers throughout the early 1990s, which, they claimed, had many other uses besides PCR operations. In 1994, the company entered into negotiations with Applied Biosystems about becoming a licensed dealer, allowing them to market their thermal cyclers for PCR [42]. After four years of fruitless talks, Applied Biosystems (through Applera) and Roche filed suit against MJ, claiming that they had been directly infringing three of the key thermal cyclers patents (US 5,333,675 ['675 patent], US 5,475,610 ['610 patent], and US 5,656,493 ['493 patent]), as well as inducing their customers to violate the three central PCR patents.

The trial did not begin until March 2004. Roche and Applera maintained that MJ Research and its two founders, brothers John and Michael Finney, had intentionally infringed the thermal cycler patents by willingly selling them to customers who they knew would use the machines for performing PCR. Several key pieces of evidence presented by Roche supporting this argument. For example, Roche and Applera showed pictures of MJ's website, taken before the trial began, in which their thermal cyclers were described as being adapted for PCR use. The websites had since been changed and the word "PCR" deleted. MJ claimed they did not know for what purposes their customers used the machines, but the plaintiffs showed a survey demonstrating that 96 percent of researchers who bought cyclers from MJ used them specifically to perform PCR reactions [42]. On April 28, 2004, the jury found MJ guilty of infringing all three of the thermal cycler patents, and inducing infringement of the PCR process patents. The jury also found that MJ and its founders had "willfully" infringed both the PCR process patents and the '493 thermal cycler patent. The jury awarded damages in the total amount of $19.8 million (see Table 1), for which MJ Research was found 90 percent liable, and John and Michael Finney each personally liable for 5 percent [43].

**Table 1:** Damages awarded in Roche v. MJ Research.

Patent	MJ Research (90%)	Michael Finney (5%)	John Finney (5%)
Process Patents	$12,474,000	$693,000	$693,000
'675	$2,673,000	$148,500	$148,500
'493	$1,603,800	$89,100	$89,100
'610	$1,069,200	$59,400	$59,400
Total Damages	$17,820,000	$990,000	$990,000
Total *Increased *Damages	$31,897,800	$1,772,100	$1,772,100

All amounts in US dollars. [43], [44]

Based on the deliberate nature of MJ's infringement, Roche and Applera sought enhanced damages. By law, the court could increase the original damages by up to three times, if the guilty party's behavior had been "reprehensible" or "egregious." As evidence of MJ's blatant guilt, Judge Janet Arterton cited testimony of past instances when MJ had given away free PCR kits when customers purchased thermal cyclers. The company had later followed up with these same customers "to both inquire whether the customers wanted another free PCR kit and to sell PCR kits [44]." Judge Arterton found sufficient misconduct to double the damages for infringement of the PCR patents and the '493 patent, the only patents which the jury had determined were "willfully" violated. In March 2005, the court increased damages from the original $19.8 million to $35.4 million, again with MJ Research responsible for 90 percent of the payment, and the Finney brothers each personally liable for 5 percent [44].

## Discussion and evaluation

While many of the patents on PCR still remain active, the expiration of the core process patents has brought the story of PCR full circle, providing a unique opportunity to reflect over the technology's effect over the past two decades. Financially, PCR has been a successful technology for its many rights-holders. In addition to evaluating financial data, it is also important to consider the role patenting and licensing of PCR have played in the dissemination of the technology in the scientific community.

### Patent expiration

On March 28, 2005, the first of the key PCR process patents expired in the United States. Reactions are mixed, with most observers undecided about the short- and long-term economic effects. Roche and the other PCR stakeholders maintain that they expect licensees to "honor the terms of their contracts", and that the losses in revenue should be kept to a minimum [45]. Despite the expirations, the companies still own the rights to dozens of PCR patents, the last of which won't expire until 2017 [15]. In addition, Roche, like other large companies such as Microsoft and Intel, is constantly modifying and upgrading its techniques and products, and should remain the key player in PCR for years to come [46]. Tom White, now the Chief Scientific Officer at Celera Diagnostics, believes that these new PCR methods, such as Real-Time PCR (RT-PCR) will ensure the technology's viability even after the core patents expire:

"Most of the ways people do PCR now is based on advances in the technology that have gone beyond the fundamental underlying patents...much more complicated versions of the process that was patented in 1987. So the fact that the fundamental patents have expired I don't think makes any difference, really...[47]"

However, many claim that the patent expirations could cut into PCR profits. In a recent quarterly report, Applied Biosystems predicted that the loss of the PCR patents could negatively impact royalty income. One San Francisco investment bank estimates that the patent expirations will cost Applied nearly $25 million annually [15].

Regardless of the impacts on PCR's parent companies, many experts in the molecular biology industry seem to believe that prices for reagents and instruments should drop, allowing PCR to spread into fields where, previously, the technique's use had been limited [45]. Environmental research and diagnostic applications are two fields where expansion in PCR use could occur. Andy Bertera of GE Healthcare claims that the effects could be felt most strongly in these areas because "cost is more of an issue...you've got a larger number of samples [45]." A drop in prices could also trigger the use of PCR into entirely new areas, such as tropical disease drug discovery, where high costs had been prohibitively expensive [46]. The reduction in costs could be rather large, claims Mark Finney, founder of Promega. He says that most companies could lower the unit price of reagent from 18 to 20 cents to just 3 to 4 cents, while still maintaining the same profit margin. He believes that reduced costs will "fuel a significant rise in the use of PCR [45]."

### Evaluating effects of intellectual property

PCR has earned huge sums of money in royalties for the various rights-holders while also becoming one of the most important research tools in molecular biology. In addition to Kary Mullis' $10,000 bonus and the $300 million earned by Cetus in selling the PCR patents, it is estimated that Roche has earned upwards of $2 billion in royalties from licensing the technology [40]. Of course, much of that money has come from the pockets of researchers, universities, and taxpayers, in the form of licensing fees, which could have been devoted to other research purposes. On the other hand, a significant portion of that revenue has gone to fueling further research and development for the companies that have controlled the patents.

When assessing the role of patenting in the development of PCR, two questions stand out: 1) did patents spur technological innovation, and 2) did the intellectual property constraints of PCR affect its dissemination? Broad patent rights and strict enforcement are necessary, many would argue, in order to provide incentives for the costly research and development of new technologies on the part of private companies. If a company cannot be assured intellectual property protection for potentially lucrative discoveries, they may be unwilling to devote significant resources into the technology in the first place. PCR presents an interesting example, since most of its more valuable applications were discovered serendipitously, not as the result of a deliberate, corporate strategy. The Cetus management was opposed to diverting resources toward the seemingly less-profitable PCR technology. So, patent protection had little bearing on the initial discovery of PCR. However, intellectual property protection *did *play an important part thereafter. While the patents did not drive the search for PCR, it did give Cetus some motivation to further the technology's development in-house. Without patent protection, and the possibility of rights to future revenue, it seems less likely that Cetus would have devoted *any *of the necessary resources that contributed to PCR's development.

Patent protection may have been essential to spur on PCR's development within Cetus, but if these same rights hinder the ability of researchers to use the technology, then the net effects might be undesirable. PCR represents a unique challenge because it is such an essential research tool, an almost ubiquitous process used in dozens of different settings in almost every branch of biology. To restrict its use could stifle discoveries that might have significant benefit for science and healthcare.

There appear to be essentially two different, but related hurdles to using PCR: the cost of performing the reaction and obtaining access to the property rights. Most complaints about access to PCR seem to focus on licensing fees and royalties (particularly with regards to the cost of *Taq *enzyme), which have made PCR prohibitively expensive in some fields. As noted above, the expiration of the core PCR patents is expected to open up new areas of research, such as environmental studies, where the costs associated with performing PCR may have made the technique unpopular. Maynard Olson, professor of genome sciences at the University of Washington, told *Science *in 2001 that "there would be a lot more genotyping done if it only cost a penny for the *Taq *[38]." A summary of a 1996 National Academy workshop on intellectual property and research tools reported that "discussion about access to PCR technology centered on the costs of *Taq *polymerase, rather than on the distribution of intellectual property rights [24]."

Despite complaints from the scientific community surrounding the cost of performing PCR, it appears as if the licensing of the technology has generally not demonstrably hindered access by researchers. PCR clearly spread throughout the scientific research community; one would be hard pressed to find a molecular biology lab in the world that does not employ the technique on a regular basis. Roche's business plans have not, apparently, prevented researchers – at least in biomedical research fields – from accessing PCR when they need it. One place it was of particular use was in DNA sequencing. Tom White, now the Chief Scientific Officer at Celera Diagnostics, points out that PCR was "used in every phase of the Human Genome Project for the storage and recovery of sequence information [48]." Tom Caskey, a past-president of the Human Genome Organization, asserted in 1997 that "if we did not have free access to PCR as a research tool, the genome project really would be undoable [24]."

**Figure 3:**
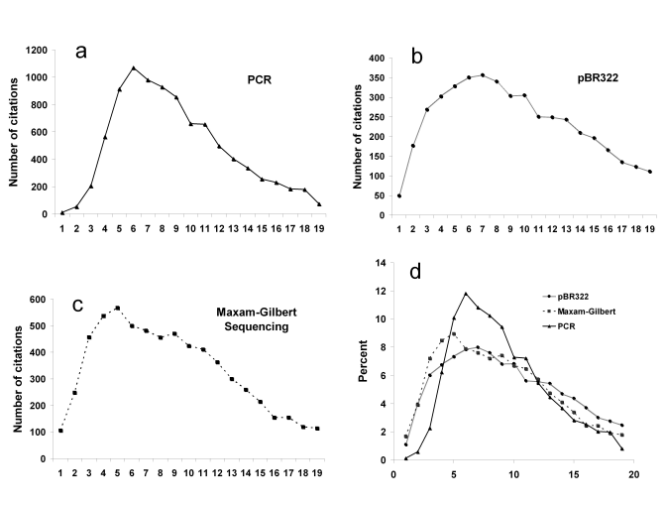
Citation trends. (a)-(c) Scientific papers citing PCR, pBR322 cloning vector, and Maxam-Gilbert sequencing by year after initial publication. Citation counts were obtained by searching Web of Science citation databases by year for articles referencing the seminal publication for each technology. Counts begin with the first full year after publication. For PCR, the citation count includes references to both the 1985 Saiki paper and the 1987 Mullis paper. (d) Normalized citation data. Y-axis represents percent of total citations in a given year for each technology. X-axis in all graphs (a-d) represents years after initial publication.

One rough metric by which to measure the adoption of a technology is to evaluate citation trends. This measure approximates the use of technology by counting the number of times publications reference the initial description of the technology in the scientific literature. Figures 3(a), 3(b), and 3(c) compare the citation trends of the two original PCR papers (Saiki et. al., 1985 and Mullis and Faloona, 1987) with the pBR322 cloning vector [49] and the Maxam-Gilbert sequencing method [50]. Both of these comparison technologies are non-patented, widely-used research tools. The qualitative similarities are apparent, a quick rise in the number of publications, a peak, then gradual decline, finally settling into a relatively constant range. To adjust for the differences in absolute numbers of citation, Figure 3d shows normalized citation data, presenting the percent of total citations in a given year for each technology.

**Figure 4:**
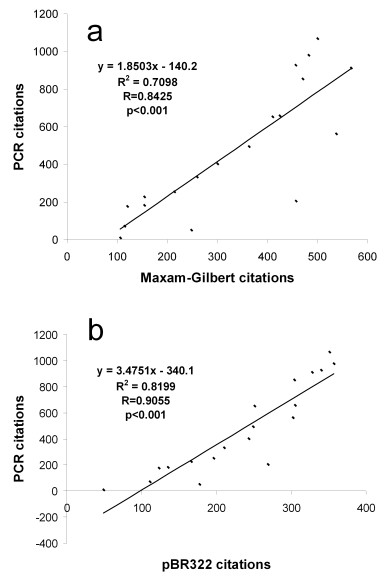
Linear correlations. (a) Parametric plot of PCR citations versus Maxam-Gilbert citations, with calculated Pearson correlation coefficient and p value. (b) Parametric plot of PCR citations versus pBR322 citations, with calculated Pearson correlation coefficient and p value.

A more quantitative approach to evaluating the citation trends can be found in Figure 4. Figure 4a shows a parametric plot of PCR citations versus Maxam-Gilbert citations, and Figure 4b shows PCR versus pBR322, with both plots using years since original publication as the independent variable. The plot of PCR versus Maxam-Gilbert has a strong positive Pearson correlation coefficient (R = 0.8425, p < 0.05), indicating a strong linear correlation between these two variables. [51] The plot of PCR versus pBR322 has an even larger correlation coefficient (R = 0.9055, p < 0.05).

Thus, in these graphs, we can see that the dissemination patterns for PCR (a patented technology with a strict, yet permissive licensing strategy) and unpatented, freely available technologies are very similar. This may suggest, at least in the case of PCR, that patenting did not severely hamper the dissemination of the technology, as compared to the dissemination of the two unpatented research tools pBR322 cloning vector and Maxam-Gilbert DNA sequencing methods. Our data does not take into account the possibility that researchers using unlicensed reagents or instruments may be less likely to cite the PCR publications if they believe that Cetus/Roche would send them a cease and desist letter. Also, our analysis cannot estimate how dissemination might have been different without a patent in the particular case of PCR.

The first year in which the number of papers citing PCR declined was 1992, the year that Roche announced the easing of licensing fees and restrictions. One would assume that the reduction in the cost of performing PCR and the ease of access to the technology would have caused a surge in the number of papers which cited PCR, encouraged by lowered prices of reagents and instruments. The fact that this did not occur suggests that the access to PCR was not unduly restricted before the lowering of fees and restrictions, and that the use of PCR in research (at least as evidenced by the citations) followed a natural pattern for similar research tools, regardless of its patent and licensing status. Citation trends appear to have a natural decline over time. This may be due to the conception that the technology is now public knowledge, which no longer requires citation, or the technology is replaced by newer or derivative technologies.

## Conclusion

While intellectual property protection was undoubtedly key to the various firms' ability to capitalize financially, these same rights did not preclude widespread adoption of the technology. The ability of PCR to become almost universally adopted while earning billions of dollars in revenue suggests that business practices that permit broad use and those that maintain profitability are not mutually exclusive. Since this example runs counter to the common assumption that strong patent rights are incompatible with widespread dissemination, it is important to examine this case history and see what factors prevented IP rights from hindering PCR's broad adoption. Based on our analysis of the history of PCR, there appear to be four key reasons why the technology's use has become so widespread, despite stringent patent protections:

• Cetus/Roche's extensive use of licensing and business partnerships,

• Fair and adaptable licensing strategies,

• Ease of obtaining licenses, and

• Widespread infringement by researchers, coupled with reluctance on the part of Cetus/Roche to sue individuals.

Early in PCR's development, a conscious and crucial decision was made on the part of Cetus to allow outside corporations to access the technology through licenses and business partnerships. This decision had tremendous consequences on the development and dissemination of the technology. The partnership with Perkin-Elmer (and later Applied Biosystems) led to the discovery of PCR as a research tool and to its automation, allowing the process to become widely available on the market and easy-to-use. The ventures with Roche and Kodak resulted in key diagnostic applications, which created a whole new market and spurred the discovery of PCR-based tests for a number of diseases. The use of nonexclusive licensing permitted broad access to PCR and prevented Cetus and/or Roche from having a monopoly on uses. Having many firms with rights to manufacture and sell reagents has led to competitive pricing, ensuring that costs are as low as they can be, subject to the obligatory royalties linked to the exclusive patent rights.

Another reason for PCR's dissemination may also have been linked to Roche's loosening of restrictions on licenses after it acquired rights to the technology. Roche appears to have responded to scientists' concerns about prohibitively high costs, and altered its licensing strategy in an attempt to encourage broad use of their technology. Tom Caskey claims that Roche "has behaved fantastically" in allowing researchers access to PCR technology [24]. Bernard Poiesz, director of the Central New York Regional Oncology Center, says that even though Roche's PCR licensing fees "are some of the highest royalty rates I have personally experienced", he knew of no other company that did a better job of making its technology available for research purposes [24].

Since each part of the PCR process – the reaction itself, the *Taq *enzyme, reagents, thermal cyclers – are all covered by separate patents, there exists the possibility that the transaction costs associated with obtaining permission and paying licensing fees did reduce some uses. Why, then, are such effects not clear-cut? One answer may lie in the ease with which licenses are obtained. Since the licenses are obtained simply by purchasing authorized instruments and reagents, there is no additional burden placed on the individual researcher beyond the higher cost caused by the patent royalty.

Walsh and colleagues have suggested another reason why patents have had little effect on the use of research tools: infringement [52]. Many researchers, either because they do not know or care, or because they have the impression that their work is protected under a "research use exemption," simply infringe the PCR patents, running reactions without purchasing licensed instruments or reagents [52]. The Promega and MJ Research legal cases demonstrate that some vendors will sell unlicensed PCR reagents at significantly lower prices than their licensed counterparts. Most of the time, researchers can continue to use the reagents with few or no repercussions. Companies have been quick to enforce their patent rights when the infringing parties are other firms, but have been "reluctant to enforce their patents against universities...because of the low damage awards and bad publicity that suing a university would entail [52]." Suits filed by firms against academic institutions are exceedingly rare. One commentator found out that aside from the *Madey v. Duke *case in 2002 (307 F 3d. 1351), she could locate only one other instance of a company suing a university for direct infringement, dating from 1967 (*John J. McMullen Associates v. State Board of Higher Education*, 268 F. Supp. 735 [D. Or. 1967]) [53].

This toleration of infringement, termed "rational forbearance" in a 1997 report by the National Research Council, has become an industry norm which seems to play a significant role in ensuring the broad use of many genomic technologies by researchers [24]. While this behavior is the norm in industry/academia relations, cases such as the DuPont oncomouse, CreLox genetic technologies, and BRCA gene patents have been viewed by many in the research community as examples of intellectual property rights-holders stifling innovative activity by excessive regulation of the uses of their products [24]. Furthermore, the *Madey v. Duke *decision in 2002, which effectively eliminated the ability of universities to hide behind the vague "research use exemption," may embolden companies to take legal action against nonprofit and academic researchers and universities. It appears that industry norms are still governing firm behavior and dissuading suits against nonprofits, but there is no way to know whether this pattern will hold, given today's legal climate. The case of PCR demonstrates one such instance in which researchers and rights-holders were able to develop an informal compromise, allowing sufficient access to the technology while maintaining its value. However, while infringement coupled with "rational forbearance" played a role in PCR's dissemination, there is some doubt as to whether researchers will be able to rely on this mechanism in the future.

Our work, while far from conclusive, seems to suggest that in the case of the polymerase chain reaction, intellectual property rights did not preclude the technology from being adopted as widely and as quickly as other, non-patented research tools. Furthermore, PCR's financial success implies that broad adoption of a platform technology does not require business practices which undermine the technology's profitability. Thus, it appears that some fears about the effects of intellectual property on research tool use can be overcome through appropriate licensing strategies and other behaviors on the part of corporate entities which control these patents.

## Competing interests

The author(s) declare that they have no competing interests.

## Authors' contributions

JF performed the majority of research and compilation of the case description and formatted the final manuscript for submission. IRW carried out the statistical analysis of citation trends and assisted with editing of all sections. BCD directed the design of the study and assisted with editing of all sections. All authors read and approved the final manuscript.
